# Enrichment of titin-truncating variants in exon 327 in dilated cardiomyopathy and its relevance to reduced nonsense-mediated mRNA decay efficiency

**DOI:** 10.3389/fgene.2022.1087359

**Published:** 2023-01-04

**Authors:** Young-gon Kim, Changhee Ha, Sunghwan Shin, Jong-ho Park, Ja-Hyun Jang, Jong-Won Kim

**Affiliations:** ^1^ Samsung Medical Center, Department of Laboratory Medicine and Genetics, Sungkyunkwan University School of Medicine, Seoul, South Korea; ^2^ Clinical Genomics Center, Samsung Medical Center, Seoul, South Korea

**Keywords:** TTN, truncating variant, exon 327, dilated cardiomyopathy, nonsense-mediated mRNA decay

## Abstract

Titin truncating variants (TTNtvs) are the most common genetic cause of dilated cardiomyopathy (DCM). Among four regions of titin, A-band enrichment of DCM-causing TTNtvs is widely accepted but the underlying mechanism is still unknown. Meanwhile, few reports have identified exon 327 as a highly mutated A-band exon but the degree of exon 327 enrichment has not been quantitatively investigated. To find the real hotspot of DCM-causing TTNtvs, we aimed to reassess the degree of TTNtv enrichment in known titin regions and in exon 327, separately. In addition, we tried to explain exon 327 clustering in terms of nonsense-mediated mRNA decay (NMD) efficiency and a dominant negative mechanism recently proposed. Research papers focusing on TTNtvs found in patients with DCM were collected. A total of 612 patients with TTNtv-realated DCM were obtained from 10 studies. In the four regions of *TTN* and exon 327, the degree of TTNtvs enrichment was calculated in a way that the effect of distribution of highly expressed exons was normalized. As a result, exon 327 was the only region that showed significant enrichment for DCM-related TTNtv (*p* < .001). On the other hand, other A-band exons had almost the same number of TTNtv of random distribution. A review of RNAseq data revealed that the median allelic imbalance deviation of exon 327 TTNtvs was .04, indicating almost zero NMD. From these findings, we propose that the widely accepted A-band enrichment of DCM-related TTNtv is mostly attributable to exon 327 enrichment. In addition, based on the recently demonstrated dominant negative mechanism, the extremely low NMD efficiency seems to contribute to exon 327 enrichment.

## Introduction

Titin-truncating variants (TTNtvs) are the most commonly observed genetic cause of dilated cardiomyopathy (DCM) and are found in up to 25% of DCM cases (Herman et al., 2012; Roberts et al., 2015; Yotti et al., 2019; Mazzarotto et al., 2020). Moreover, approximately 1% of the general population carry TTNtvs, necessitating the parameters for TTNtv pathogenicity prediction (Roberts et al., 2015; Schafer et al., 2017; Yotti et al., 2019). Percent spliced-in (PSI) has been reported as a crucial pathogenicity predictor of TTNtv in DCM. Clinical data demonstrated that TTNtvs located in exons that are constitutively expressed in the human left ventricle (PSI > .9) are significantly associated with DCM (Roberts et al., 2015; Schafer et al., 2017; Haggerty et al., 2019). In addition to PSI, the location of premature termination codon (PTC) has also been investigated as a parameter for pathogenicity prediction of TTNtv. As a result, among the four protein regions of titin, i.e., Z-disk, I-band, A-band, and M-band, A-band enrichment of TTNtv in patients with DCM has been repeatedly reported (Herman et al., 2012; Roberts et al., 2015; Akinrinade et al., 2016; Schafer et al., 2017; Rich et al., 2020; Fomin et al., 2021). TTNtvs from all titin regions can lead to DCM, and A-band TTNtvs are known to have a higher odds ratio (OR) than other regions (Akinrinade et al., 2016; Schafer et al., 2017). The mechanism of A-band enrichment remains unclear. Meanwhile, in few reports, exon 327 within the A-band has been briefly mentioned as a frequently mutated *TTN* exon (Akinrinade et al., 2016; Tharp et al., 2019; Fomin et al., 2021). The degree of TTNtv enrichment in exon 327 and other A-band exons has not been separately analyzed.

The A-band is the longest region of *TTN*, which comprises 49.8% (53,799 bp/1,07,976 bp) of the *TTN* coding region, and when limited to high-PSI exons (PSI > .9), the A-band accounts for 67.0% (53,799 bp/80,325 bp) of the coding region. Considering the high share A-band holds in the high-PSI exons, a high frequency of DCM-related TTNtv in the A-band is expected. In this study, we aimed to assess the degree of TTNtv enrichment in the titin regions when the effect of the distribution of high-PSI exons was removed. Additionally, we aimed to measure the degree of TTNtv enrichment in the exon 327 and other A-band exons separately.

As a disease mechanism of TTNtv-related DCM, in addition to haploinsufficiency caused by the shortage of full-length titin proteins, a dominant negative effect caused by truncated titin protein has been demonstrated in recent reports (Fomin et al., 2021; McAfee et al., 2021). In this scenario, evasion of nonsense-mediated mRNA decay (NMD) is necessary to create a truncated titin protein that acts as a poison peptide. Because the amount of truncated titin protein has been demonstrated to affect the outcome of DCM (Fomin et al., 2021), a reasonable assumption is that NMD efficiency may affect the pathogenesis of TTNtv in DCM.

Exon 327 is the longest exon in *TTN*—by far—consisting of 17,106 bp (median = 261 bp, second-longest exon = 5,609 bp; Supplementary Table S1). According to reports on the emerging parameters of NMD efficiency, PTCs in long exons tend to decrease the efficiency of NMD because of the long average distance from the PTC to the downstream intron (PTC-to-intron distance). A long PTC-to-intron distance hinders the contact between PTC-bound ribosomes and the downstream exon-junction complex, which is required for NMD initiation (Lindeboom et al., 2016; Hoek et al., 2019; Supek et al., 2021). The association of a large PTC-to-intron distance and inefficient NMD was recorded in a study that utilized single-molecule imaging (Hoek et al., 2019). In addition, *TTN* was reported to be one of the genes enriched for pathogenic truncating variants that do not trigger NMD (Lindeboom et al., 2019). Our third aim was to identify whether the exon 327 enrichment of TTNtv in patients with DCM is the consequence of reduced NMD efficiency caused by long PTC-to-intron distance. Lastly, we also aimed to determine whether there is a correlation between NMD efficiency and clinical outcomes of TTNtv-related DCM.

### Data acquisition

This study was based on a literature review and was thus exempt from any local institutional ethics board review. We searched Google Scholar and PubMed for papers in the English language from January 2010 to May 2022 for the terms “titin,” “truncating variant,” and “dilated cardiomyopathy.” A list of the included studies is presented in Table 1. Tayal et al. (2017) was excluded from the analysis because the study was performed in the same hospital as Roberts et al. (2015) and there was significant overlap in the study period and the list of variants. In addition, among the cases of Jansweijer et al. (2017), cases from the University Medical Center Groningen were excluded because Jansen et al. (2019) also retrospectively utilized DCM probands from the same hospital, and there was significant overlap in the study period and list of variants. Similarly, among the cases of Herman et al. (2012), those from Royal Brompton and Harefield Hospitals NHS Foundation Trust were excluded because of the overlap with Roberts et al. (2015) in hospital, study period, and variants.

**TABLE 1:** List of research papers utilized and the number of TTNtvs from each study.

Study	Country	DCM patient enroll years	No. of DCM probands	No. of DCM probands with TTNtv (PSI > .9)			No. of cases utilized in this study		
				Frameshift	Non-sense	Total	Regional enrichment analysis	RNAseq data analysis	Phenotype analysis
Ahktar et al. (2020)	13 European countries, Australia	1985–2019	N/A	122 (121)	158 (158)	280 (279)	279		
Fomin et al. (2021)	Germany	Published 2021	113	9 (9)	12 (12)	21 (21)	21	11	21
Franaszczyk et al. (2017)	Poland	2012–2014	72	7 (7)	10 (10)	17 (17)	17		
^a^Herman et al. (2012)	United States, United Kingdom, Italy	Published 2012	203	12 (11)	22 (22)	34 (33)	19		33
Jansen et al. (2019)	Netherlands	2012–2016	N/A	59 (58)	55 (54)	114 (112)	112		
^b^Jansweijer et al. (2017)	Netherlands	2012–2014	133	9 (9)	9 (9)	18 (18)	18		
^c^McAfee et al. (2021)	United States	Published 2021	184	N/A	N/A	22 (22)		19	22
Pugh et al. (2014)	United States	2007–2012	156	12 (12)	4 (4)	16 (16)	16		
Rich et al. (2020)	United States	2014–2018	N/A	12 (12)	13 (12)	25 (24)	24		
Roberts et al. (2015)	United Kingdom	1993–2011	529	33 (32)	32 (32)	65 (64)	64	12	
Total				275 (271)	315 (313)	612 (606)	570	42	76

^a^ Cases from Royal Brompton and Harefield Hospitals NHS Foundation Trust was excluded from regional enrichment analysis because of the redundancy of cases with Roberts et al., 2015.

^b^ Cases from University Medical Center Groningen was excluded because of the redundancy of cases with Jansen et al., 2019.

^c^ Premature termination codon location, allelic imbalance deviation, age of heart transplantation, and left ventricular ejection fraction were utilized as provided without full variant description.

TTNtv, titin-truncating variant; DCM, dilated cardiomyopathy; PSI, percent spliced in; N/A, not available.

Patients with *TTN* nonsense variants or frameshift variants were selected from the final included papers. Patients with splice-site variants were excluded because their exact PTC location could not be determined. Exceptionally, in RNAseq data analysis, splice-site variants could not be filtered out and all truncating variants were included because in one study (McAfee et al., 2021), the allelic imbalance (AI) deviation values were given without specific variant description. Cases with frameshift variants with incomplete descriptions for exact PTC location determination (e.g., p.Ser4228Leufs instead of p.Ser4228LeufsTer23 without cDNA description) were also excluded. The variants described according to the transcripts other than the meta-transcript (NM_001267550.2) were transformed to NM_001267550.2 using VariantValidator (Freeman et al., 2018). (https://variantvalidator.org), and the PTC location was determined based on the amino acid sequence according to NP_001254479.2. Exon number, length of PTC-containing exon, and PTC-to-intron distance were annotated using the exon boundary information downloaded from the Table Browser of the UCSC genome browser (Supplementary Table S1) (Karolchik et al., 2004). PSI and titin protein regions were annotated using the data of Roberts et al. (2015).

The gnomAD v2.1.1 data (Karczewski et al., 2020) was used as a control group, and a list of *TTN* variants was downloaded from the official gnomAD website (https://gnomad.broadinstitute.org/). Splice-site variants were removed; the annotation of PTC location, exon, and region information, and PSI was performed as aforementioned.

### TTNtv enrichment only in exon 327 in DCM group

**FIGURE 1:**
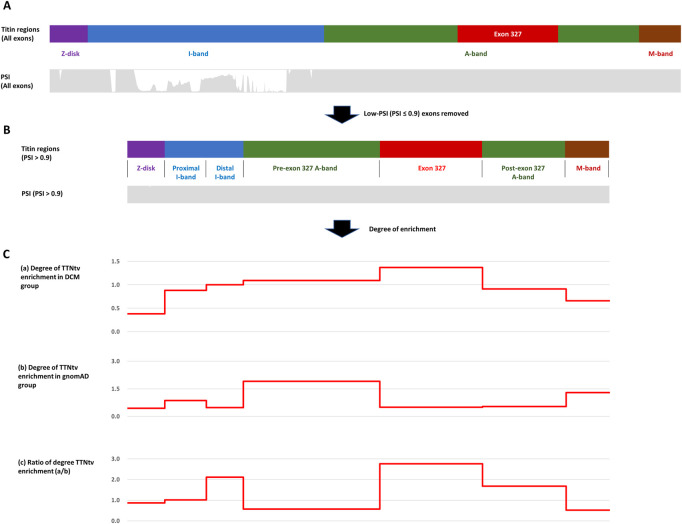
**(A)** Four regions of titin meta-transcript (NM_001267550.2) with PSI (Percent spliced-in) distribution. **(B)** After low-PSI exons were removed, I-band was divided into two sub-regions (Proximal I-band: exon 29–50, Distal I-band: exon 220–252), and A-band was divided into three sub-regions (Pre-exon 327: exon 253–326, exon 327, post-exon 327: exon 328–358). **(C)** Degree of Titin-truncating variant (TTNtv) enrichment calculated based on the ratio of observed and expected (based on the length of the region) number of TTNtv. **(A)** DCM group **(B)** gnomAD group **(C)** DCM group/gnomAD group.

For known titin protein regions and exon 327, the degree of TTNtv enrichment was calculated based on the ratio of the observed and expected number of TTNtvs (expected based on the length of regions) in both the DCM and control groups. To remove the effect of PSI, a confounding variable in measuring the effect of PTC location on the pathogenesis of DCM, we utilized only high-PSI exons (PSI > .9) both for collecting TTNtvs and for calculating the coding region length (Figure 1). After removing low-PSI regions, the I-band was divided by the major splicing region, resulting in two sub-regions, the proximal I-band and distal I-band, as in other studies (Schafer et al., 2017; Fomin et al., 2021). To focus on the degree of TTNtv enrichment in exon 327, we also divided the A-band into three sub-regions: pre-exon 327 A-band, exon 327, and post-exon 327 A-band. For determining the degree of TTNtv enrichment in the DCM group compared with the control group, the ratio of the degree of TTNtv enrichment in the DCM group to that in the control group was derived.

From 10 cohort studies, 612 cases of TTNtv-related DCM were found (Table 1; Supplementary Table S2) (Herman et al., 2012; Pugh et al., 2014; Roberts et al., 2015; Franaszczyk et al., 2017; Jansweijer et al., 2017; Jansen et al., 2019; Akhtar et al., 2020; Rich et al., 2020; Fomin et al., 2021; McAfee et al., 2021). Among these cases, 606 had variants in the high-PSI (>.9) exons. Except for the 22 cases in which full variant descriptions were not available (McAfee et al., 2021) and 14 cases excluded because of redundancy (Herman et al., 2012), 570 cases were used in the regional enrichment analysis constituting the *DCM group*. The number of cases utilized in the RNAseq data analysis and phenotype analysis were 42 and 76, respectively (Table 1; Supplementary Tables S3, S4). From gnomAD v2.1.1, which consisted of 141,456 cases, 1,749 cases (1.24%) with TTNtv were found (Supplementary Tables S5, S6). After removing variants from low-PSI exons and terminal exon of novex-3 (ENST00000360870.5) that cannot be converted to the meta-transcript (Roberts et al., 2015), 957 cases (0.68%) remained and were used in the regional enrichment analysis as a control group (Supplementary Tables S6, S7).

**FIGURE 2:**
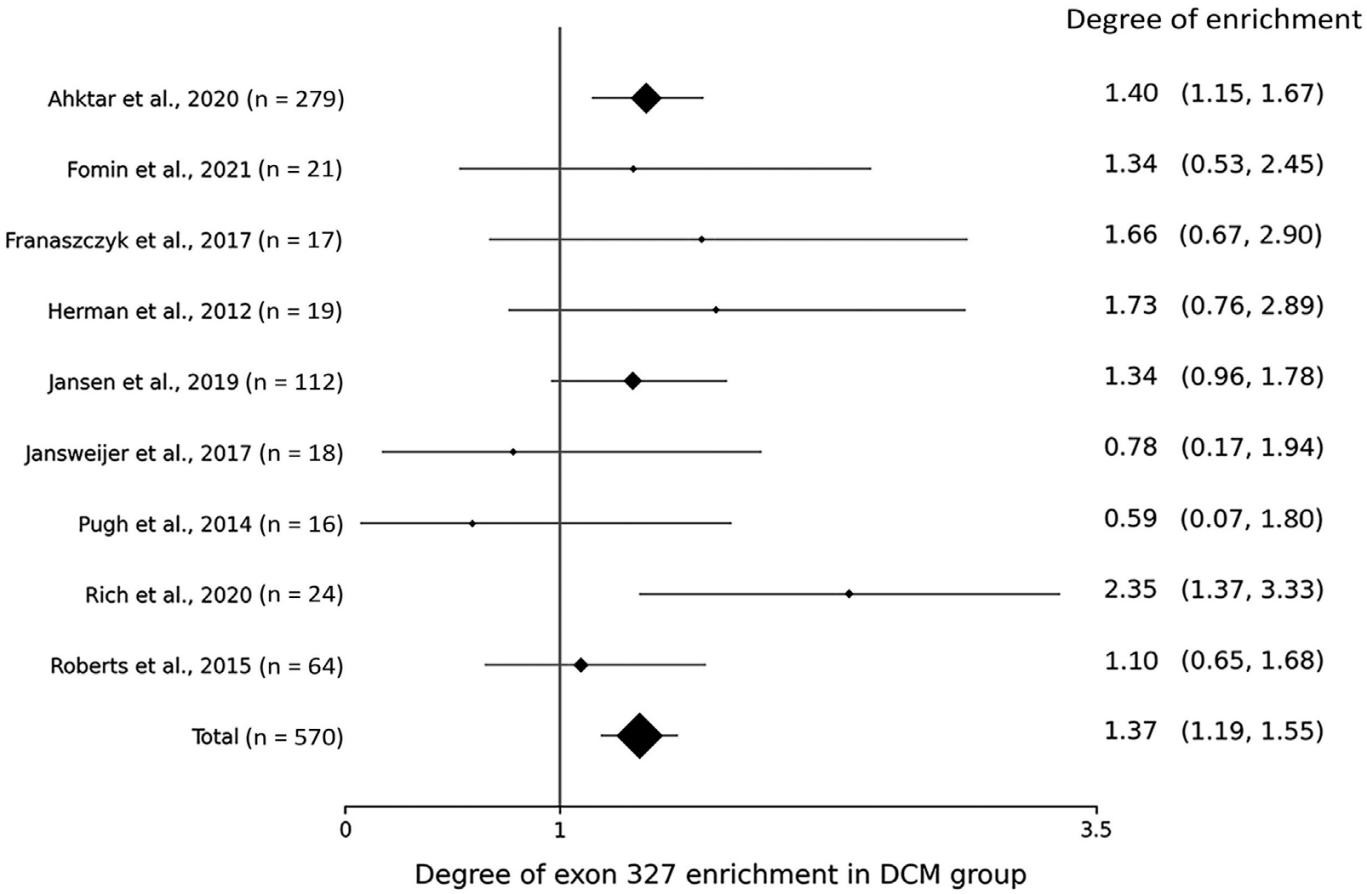
Forest plot showing the degree of titin-truncating variant (TTNtv) enrichment in exon 327 from individual studies.

Within the DCM group, the degree of TTNtv enrichment was the highest in exon 327 (1.37, *p* < .001) (Table 2; Figure 1). The enrichment of total A-band TTNtvs was statistically significant (*p* < .001); however, after excluding exon 327 TTNtvs, the degree of enrichment in the A-band did not show a significant increase in either pre-exon 327 or post-exon 327 TTNtvs (*p* = .178 and *p* = .347, respectively). The degree of I-band TTNtv enrichment was also non-significant, with a value close to 1.0 (.94, *p* = .533). The degree of Z-disk and M-band enrichment was below 1.0 (.38 and .66, respectively), which was statistically significant (*p* < .001 and *p* = .010, respectively). When separated into individual studies, statistically significant enrichment in exon 327 was observed only in two studies (Akhtar et al., 2020; Rich et al., 2020), presumably due to the smaller sample sizes (Figure 2).

**TABLE 2:** Degree of TTNtv enrichment in DCM group and in gnomAD calculated based on the ratio of observed and expected number of TTNtv from exons with PSI > .9.

Region	Coding length (Total = 80,325)	% Coding length	DCM group (N = 570)				gnomAD group (N = 957)				DCM group vs. gnomAD group	
			(a) No. of TTNtv observed	(b) No. of TTNtv expected	(c) Degree of TTNtv enrichment (a/b)	*^a^* *p*-value	(d) No. of TTNtv observed	(e) No. of TTNtv expected	(f) Degree of TTNtv enrichment (d/e)	*^a^* *p*-value	Ratio of degree of TTNtv enrichment (c/f)	^b^ *p*-value
Z-disk	6,232	7.76	17	44.22	.38	<.001	33	74.25	.44	<.001	.86	.729
I-band	13,083	16.29	87	92.84	.94	.533	106	155.87	.68	<.001	1.38	.021
Proximal	6,912	8.61	43	49.05	.88	.411	71	82.35	.86	.205	1.02	1.000
Distal	6,171	7.68	44	43.79	1.00	.937	35	73.52	.48	<.001	2.11	<.001
A-band	53,799	66.98	432	381.77	1.13	<.001	707	640.97	1.10	<.001	1.03	.442
Exon 327	17,106	21.30	166	121.39	1.37	<.001	101	203.80	.50	<.001	2.76	<.001
Other exons	36,693	45.68	266	260.38	1.02	.644	606	437.16	1.39	<.001	.74	<.001
Pre-exon 327	22,749	28.32	176	161.43	1.09	.178	516	271.03	1.90	<.001	.57	<.001
Post-exon 327	13,944	17.36	90	98.95	.91	.347	90	166.13	.54	<.001	1.68	<.001
M-band	7,211	8.98	34	51.17	.66	.010	111	85.91	1.29	.007	.51	<.001

Low-PSI (PSI ≤ .9) exons were removed from analysis, and the coding length and number of TTNtv were derived only from high-PSI (PSI > .9) exons.

^a^ *p*-values were derived from the comparison of the two proportions, % coding length and % TTNtv, using the binomial test.

^b^ *p*-values were derived from the comparison of the two proportions, %TTNtv in DCM group and in gnomAD group, using the binomial test.

TTNtv, titin-truncating variant; DCM, dilated cardiomyopathy; gnomAD, genome aggregation database; PSI, percent spliced-in.

From the gnomAD group, while the degree of TTNtv enrichment showed considerable fluctuation, the degree of enrichment exon 327 was low (.50, *p* < .001) reflecting deprivation of exon 327 TTNtv in general population (Table 2; Figure 1). When the degree of enrichment in the DCM group was divided by that of the gnomAD group, the ratio was the highest in exon 327 among all regions of *TTN* (2.76, *p* < .001).

### Generally reduced NMD efficiency especially in exon 327

In the three studies in which RNAseq was performed (Roberts et al., 2015; Fomin et al., 2021; McAfee et al., 2021), AI deviation was obtained as a surrogate for the actual NMD efficiency such that 0 and .5 indicate no NMD at all and 100% NMD efficiency, respectively. AI deviation was directly provided in one study (McAfee et al., 2021), and in two other studies (Roberts et al., 2015; Fomin et al., 2021), AI deviation was derived from altered allele frequency (altered AF) simply by subtracting the altered AF from .5. A value of zero was used as the AI deviation when a negative value was obtained. From the three studies, PTC location, age at heart transplantation (HT), and left ventricular ejection fraction (LVEF) were also obtained from the available cases, and the correlations among the four variables (AI deviation, PTC location, age at HT, and LVEF) were analyzed.

**FIGURE 3:**
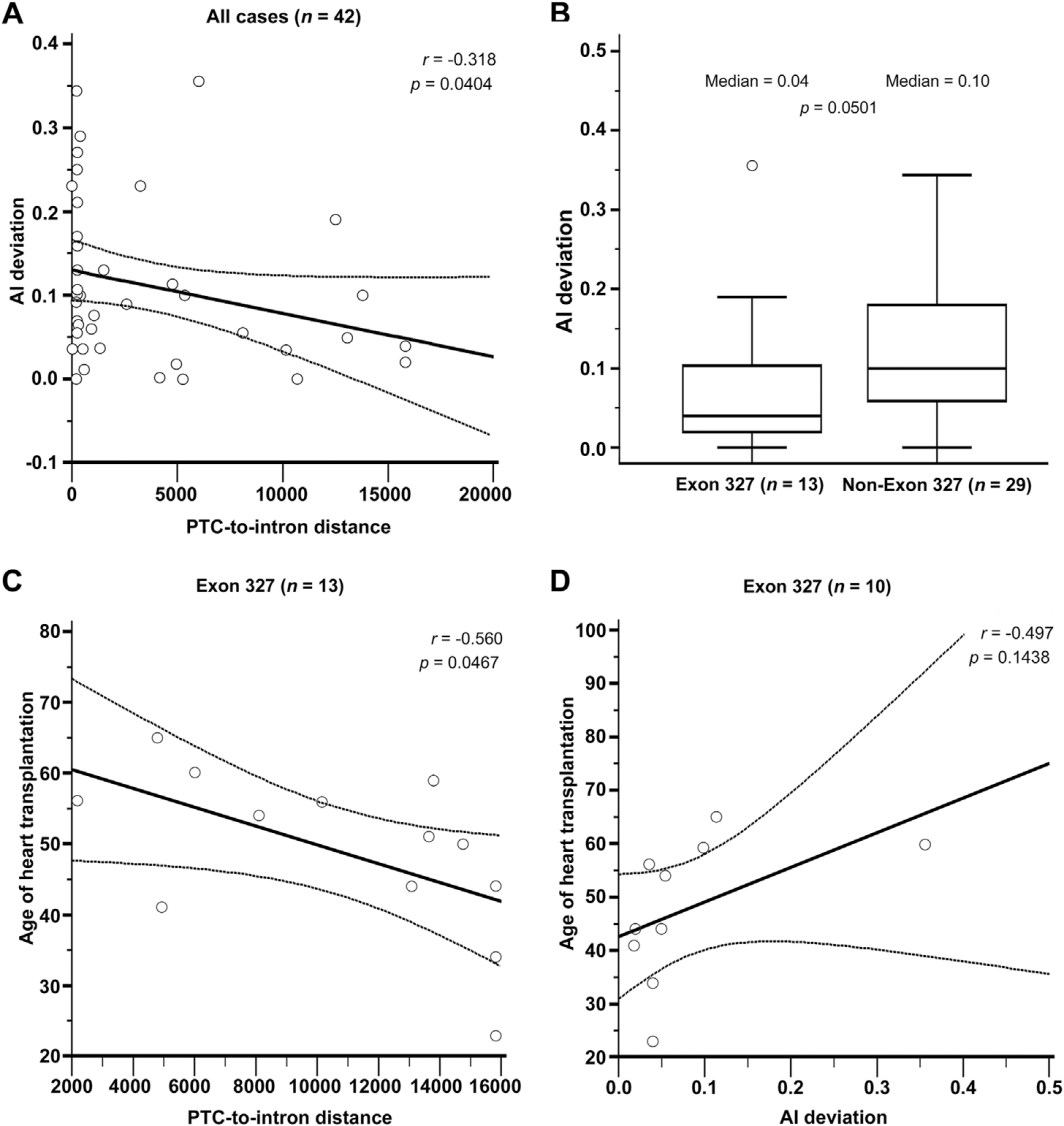
**(A)** Relationship between Premature termination codon (PTC)-to-intron distance and allelic imbalance deviation (AI deviation). **(B)** Difference in nonsense-mediated mRNA decay efficiency measured by AI deviation level between exon 327 and non-exon 327 titin-truncating variants (TTNtvs). Median PTC-to-intron distance was 10,693 and 223 in exon 327 group and non-exon 327 TTNtvs, respectively (*p* < .001). **(C)** Relationship between PTC-to-intron distance and age of heart transplantation in carriers of exon 327 TTNtv. **(D)** Relationship between AI deviation and age of heart transplantation in carriers of exon 327 TTNtvs. *p*-values are from Pearson’s correlation test and the Mann–Whitney test. PTC, premature termination codon; AI, allelic imbalance; TTNtv, titin-truncating variant.

The results of RNAseq data analysis and phenotype analysis are shown in Figure 3. The PTC-to-intron distance was negatively correlated with allelic imbalance (AI) deviation (*r* = .318, *p* = .0404, Figure 3A). The level of NMD measured using AI deviation was low in most DCM cases. The median AI deviation was higher in non-exon 327 TTNtvs than in exon 327 TTNtvs, but the difference was non-significant (median AI deviation of .04 and .10, respectively, *p* = .050, Mann–Whitney U test, Figure 3B). When limited to exon 327 TTNtvs, PTC-to-intron distance was negatively correlated with age at HT (*r* = −.560, *p* = .0467, Figure 3C). AI deviation also showed a similar effect on the age at HT, but without statistical significance, probably because of the reduced sample size (*r* = −.497, *p* = .1438, Figure 3D). Other correlation analyses among available parameters, PTC-to-intron distance, AI deviation, age at HT, and LVEF, including subgroup analysis (exon 327 and non-exon 327 TTNtvs), did not show any statistical significance (Supplementary Figure S1).

### Phenotype analysis

Utilizing studies in which clinical outcome data were provided and the data could be linked to specific TTNtvs (Herman et al., 2012; Fomin et al., 2021; McAfee et al., 2021), clinical outcomes between carriers of exon 327 TTNtv and non-exon 327 TTNtv were compared (Table 3). Seven clinical indicators, including diagnosis age, LVEF, and family history, were used; however, statistical significance was not observed for all parameters.

**TABLE 3:** Phenotype comparison of exon 327 TTNtv and non-exon 327 TTNtv.

Clinical indicator	Available cases (N)	Exon 327	Non-exon 327	*p*-value
Diagnosis age	33	37 (33–45)	37 (29–49)	0.6879^b^
LVEF	76	20 (15–29)	20 (15–29.75)	
Fractional shortening	17	14.3 (13.4–16.3)	18.45 (11.0–22.4)	0.5362
LVEDD	32	65 (51–77)	64 (61–73)	0.9351^b^
NYHA class	34	3 (2–4)	3.25 (3–4)	0.4282
Age of endpoint^a^	63	51 (41–55.75)	50.5 (39–57.5)	0.9343
Family history	30	9	21	0.2096
Yes		8	13	
No		1	8	

Values expressed as median (IQR).

^a^ Endpoint was defined as heart transplantation, death, or ventricular assist device insertion.

^b^ *t*-test for normally distributed continuous variables.

*p*-values were obtained from Mann–Whitney tests for non-normally distributed continuous variables.

Fisher’s exact tests for categorical variables.

TTNtv, titin-truncating variant; LVEF, left ventricular ejection fraction; LVEDD, left ventricular end-diastolic dimension; NYHA, New York heart association.

### Statistical analysis

The Shapiro test was used to test the normality of the data. For the comparison of the two groups, the *t*-test was used when the normality assumption was satisfied; otherwise, the Mann–Whitney test was used. Pearson’s correlation was used to analyze the correlation between two numerical variables when the normality assumption was satisfied, and Spearman’s correlation analysis was used otherwise. The Fisher’s exact test was used to compare categorical variables between groups and binomial test was used for the comparison of proportions. Shapiro test and binomial test were performed using R (version 4.0.3) software and all other tests were performed using MedCalc Statistical Software version 19.2.6 (MedCalc Software Ltd., Ostend, Belgium; https://www.medcalc.org;2020).

## Discussion

Studies have reported that TTNtvs that affect high-PSI (>.9) exons have a 93% probability of being pathogenic, and for A-band TTNtvs, the probability is higher, at 97% (Roberts et al., 2015; Akinrinade et al., 2016). From case control studies, A-band TTNtv showed an OR of 49.8–70.4, and TTNtv from other regions showed an OR of 5.3 (Z-disk), 3.7 (M-band), 19.0 (proximal I-band), and 19.5–32.0 (distal I-band), when limited to constitutive exons (Akinrinade et al., 2016; Schafer et al., 2017). In our study, the total number of patients with DCM (with or without TTNtv) was not available in three studies (Table 1) (Jansen et al., 2019; Akhtar et al., 2020; Rich et al., 2020), making it impossible to derive OR. Instead, we analyzed the degree of enrichment based on the ratio of the observed and expected numbers of TTNtv (expected based on the length of the high-PSI coding region). The degree of TTNtv enrichment among titin regions and exon 327 could be quantitatively compared.

Although exon 327 has been designated as a potential hotspot in several reports (Akinrinade et al., 2016; Tharp et al., 2019; Fomin et al., 2021), the degree of enrichment of exon 327 and other A-band exons has not been separately analyzed. In the DCM cohorts in our study, the number of TTNtv observed in exon 327 was 37% higher than the expected number based on uniform distribution (*p* < .001), and no other regions showed a significant increment or increment of >10%. The non-exon 327 A-band showed a minimally (2%) higher number of TTNtv than that with the uniform distribution, which was statistically non-significant (*p* = 0.644). Overall, except for exon 327, the number of DCM-related TTNtv was almost the same as the expected number of uniform distributions in the A-band and I-band, whereas that of the Z-disk and M-band was significantly lower. Based on this result, the widely accepted A-band enrichment appears to be mostly attributable to exon 327.

In contrast with the DCM cohorts, in which the fluctuation in the degree of TTNtv enrichment was not substantial and consistent with the existing knowledge except for the exon 327 vs. other A-band exons, in the gnomAD cohort, the degree of TTNtv enrichment showed considerable fluctuation in an unpredicted manner. Although the allele frequency (AF) of a variant in the general population is known to reflect the severity of the phenotype by the purifying selection pressure (Gorlov et al., 2011), this might not work in a precise, quantitative way for late-onset, incompletely-penetrant phenotypes, such as *TTN*-related DCM. A high proportion of Clinvar (likely) pathogenic variants (21.6% among high-PSI variants, 12.1% among all variants, Supplementary Table S8) among the TTNtv included in gnomAD cohort indicates that the control group may not consist of individuals with a purely normal cardiac phenotype. Although exon 327 remained the most significantly enriched area when both DCM and gnomAD cohorts were considered, we posit that the results from DCM cohorts are a more direct reflection of the status of TTNtv distribution in patients with DCM.

The pathogenic mechanism of TTNtv leading to DCM is not well understood. It was once proposed that Cronos titin isoform, which encodes the most distal region of the I-band, the A-band and the M-band, can rescue the phenotype explaining the A-band enrichment (Zou et al., 2015; Schafer et al., 2017; Santiago et al., 2021). However, TTNtvs located in pre-Cronos regions also showed high OR (Schafer et al., 2017) and the absence of compensatory changes in Cronos isoform was demonstrated (Fomin et al., 2021), contradicting the hypothesis. The debate between haploinsufficiency and a dominant negative mechanism is ongoing (Santiago et al., 2021) and despite the clues for dominant negative mechanisms, such as the tendency of higher pathogenicity of more distally located TTNtv (Roberts et al., 2015), direct evidence had not been observed until the existence of truncated titin proteins was proved in affected human heart tissue in two recent studies (Fomin et al., 2021; McAfee et al., 2021). In these two reports, truncated titin protein was quantitatively detected by methods, such as Western blotting. Truncated titin proteins were present in intracellular aggregates rather than sarcomeres (Fomin et al., 2021), and the sizes of truncated proteins corresponded to that predicted by the location of TTNtvs (McAfee et al., 2021). Disease severity did not correlate with TTNtv location, but the level of truncated titin protein negatively correlated with patient age at HT (Fomin et al., 2021). These two reports suggested a multifactorial mechanism of combined dominant-negative and haploinsufficiency.

**FIGURE 4:**
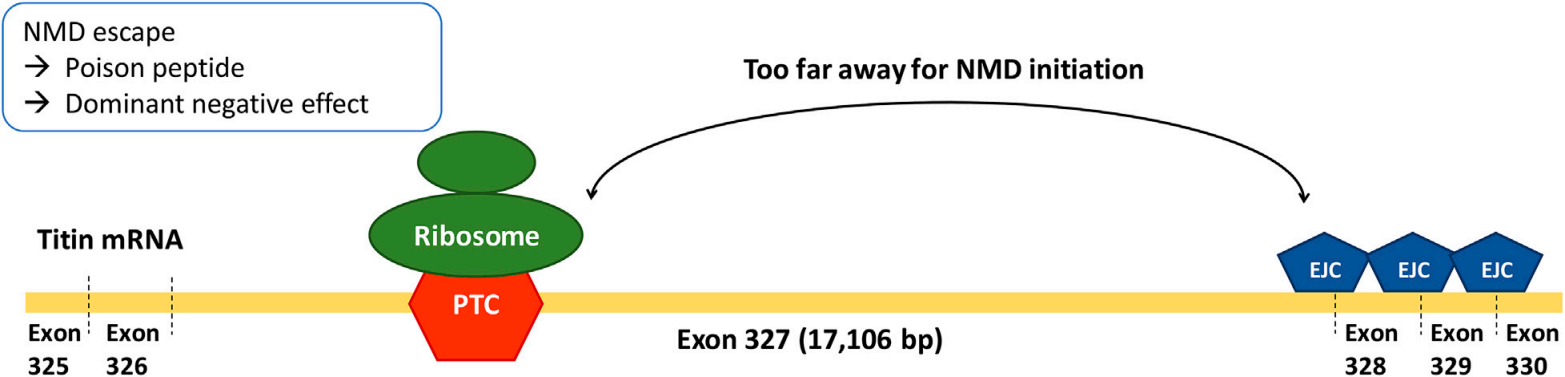
Long PTC-to-intron distance of Exon 327 TTNtvs leading to extremely low NMD efficiency.

In the RNAseq data analysis, a negative correlation between PTC-to-intron distance and AI deviation was observed, confirming the negative effect of long PTC-to-intron distance on NMD efficiency in the literature (Hoek et al., 2019; Supek et al., 2021). DCM-related TTNtv showed generally reduced NMD efficiency (median AI deviation = .10), and exon 327 TTNtv showed almost no NMD (median AI deviation = .04). Although the difference between exon 327 TTNtv and other DCM-related TTNtv did not reach statistical significance (*p* = .0501), the low AI deviation values generally observed for DCM-related TTNtvs demonstrated the importance of NMD evasion in the pathogenesis of TTNtv-related DCM. This result is in concordance with a report in which *TTN* was included in the list of genes enriched for pathogenic truncating variants that do not trigger NMD (Lindeboom et al., 2019). The extremely low NMD efficiency of exon 327 TTNtvs, presumably caused by the long PTC-to-intron distance, seems to be a cause of the high frequency of exon 327 TTNtvs observed in patients with DCM (Figure 4).

There was no significant difference in the clinical outcome parameters between patients with exon 327 TTNtvs and those with other TTNtvs. However, when limited to exon 327 TTNtvs, the age at HT showed a negative correlation with PTC-to-intron distance, indicating that extremely reduced NMD efficiency may affect the clinical outcome. However, whether predicting the severity of DCM based on the PTC-to-intron distance of TTNtv is possible remains unclear.

Although NMD efficiency seems to explain certain levels of the pathogenicity of TTNtv, especially in exon 327, other factors might contribute to its pathogenesis. The level of truncated titin protein should have a more direct effect on penetrance and pathogenicity than the mRNA level. Considering the observation that decreased NMD efficiency did not lead to a proportionally increased production of truncated proteins (Fomin et al., 2021), factors other than NMD might regulate the production and degradation of truncated proteins. Because of the small sample size used in the study that negated the direct relationship between NMD efficiency and protein levels (*n* = 11) (Fomin et al., 2021), further investigation of the regulation of mRNA and protein levels and its effect on clinical outcomes is warranted.

In conclusion, the accepted A-band enrichment of DCM-related TTNtv was mostly attributable to exon 327 enrichment. The extremely low NMD efficiency of exon 327 TTNtvs, presumably caused by the long PTC-to-intron distance, might cause the high frequency of exon 327 TTNtvs observed in patients with DCM. Reduced NMD efficiency may play an important role in the pathogenesis of TTNtv-related DCM, particularly in exon 327.

## Data Availability

The original contributions presented in the study are included in the article/Supplementary Material, further inquiries can be directed to the corresponding author.
