# Supplementary material for: Enrichment of titin-truncating variants in exon 327 in dilated cardiomyopathy and its relevance to reduced nonsense-mediated mRNA decay efficiency
Source: Front Genet. 2023 Jan 4;13:1087359. doi: 10.3389/fgene.2022.1087359 (PMC9845391; doi:10.3389/fgene.2022.1087359)
Supplement: Supplementary file 2 [file Table1.DOCX]

Supplementary Material





**Supplementary Figure S1.** Analysis of correlation among four variables from RNAseq data analysis. Statistically significant correlation was not observed except for the pairs presented in Figure 3 of the main article
